# Diagnostic Performance of Self-Assessment for Constipation in Patients With Long-Term Opioid Treatment

**DOI:** 10.1097/MD.0000000000002227

**Published:** 2015-12-18

**Authors:** Sascha Tafelski, Felicitas Bellin, Claudia Denke, Torsten Beutlhauser, Thomas Fritzsche, Christina West, Michael Schäfer

**Affiliations:** From the Department of Anaesthesiology and Intensive Care, Charité-Universitaetsmedizin Berlin, Campus Charité Mitte and Campus Virchow-Klinikum, Augustenburger Platz 1, Berlin, Germany.

## Abstract

Constipation is a prevalent comorbidity affecting ∼50% of patients with long-term opioid therapy. In clinical routine different diagnostic instruments are in use to identify patients under risk. The aim of this study was to assess the diagnostic performance of an 11-item Likert scale for constipation used as a self-assessment in opioid-treated patients.

This trial was conducted as a retrospective cohort study in Berlin, Germany. Patients with long-term opioid therapy treated in 2 university-affiliated outpatient pain facilities at the Charité hospital were included from January 2013 to August 2013. Constipation was rated in a self-assessment using a numeric rating scale from 0 to 10 (Con-NRS) and compared with results from a structured assessment based on ROME-III criteria.

Altogether, 171 patients were included. Incidence of constipation was 49% of patients. The receiver-operating characteristic of Con-NRS achieved an area under the curve of 0.814 (AUC 95% confidence interval 0.748–0.880, *P* < 0.001). Con-NRS ≥ 1 achieved sensitivity and specificity of 79.7% and 77.2%, respectively. The positive predictive value and the negative predictive value were 70.3% and 81.6%, respectively.

Overall diagnostic performance of a concise 11-item Likert scale for constipation was moderate. Although patients with long-term opioid therapy are familiar with numeric rating scales, a significant number of patients with constipation were not identified. The instrument may be additionally useful to facilitate individualized therapeutic decision making and to control therapeutic success when measured repetitively.

## INTRODUCTION

In a large observational study in the USA altogether 18.9% of patients reported on symptoms of constipation during the preceding year.^1^ Opioid intake increased the odds for constipation by factor 1.6 representing the most important side effect of these drugs.^2^ Affected patients experience a significant impairment in quality of life ^3^ and constipation is one of the main reasons to change prescribed therapy.^4–6^ Focusing patients with long-term opioid therapy, the incidence of constipation reaches ∼30% to 50% and a number-needed-to-harm of 3.4 was described.^2,7^

The current gold standard for diagnosing functional constipation is using ROME–III criteria.^8^ Accordingly, patients are required to assess only frequency of defecations (<3 per week), straining during defecation, lumpy or hard stools, sensations of incomplete evacuation or of anorectal obstruction and, whether manual maneuvers are required. With 2 or more of these criteria functional constipation is diagnosed. Also, the need for laxatives to achieve loose stool is incorporated into these criteria.^8^

Even though gastrointestinal symptoms are common side effects of opioids, it might be conflicting for patients to report symptoms. Consequently, alternative tools were developed to support diagnosing of constipation.^9^ Therefore, simple and reliable screening instruments were searched.^10,11^ Patients receiving opioid medication are very familiar with the use of Likert scales like the numeric analogue scale for pain. This scale has also been validated to assess other symptoms such as nausea ^12^ and is part of validated standardized screening questionnaires for anxiety and depression.^13,14^ Similarly, an 11-item version of the scale was described to assess constipation. This scale was validated in patients with advanced cancer by Rhondali et al and in a sample of palliative patients by Noguera et al.^15,16^ In both studies, diagnostic test performance was sufficient with sensitivity >80% but a significant number of patients were not detected. However, this 11-point Likert screening instrument for constipation has not yet been validated in a general population of patients with long-term opioid therapy.

Therefore, this study aimed to evaluate the accuracy and diagnostic test characteristics of the screening instrument for opioid-induced constipation.

## MATERIALS AND METHODS

This study was performed as a retrospective cohort study including all consecutive patients presenting in 2 hospital-affiliated specialized outpatient pain centers in Berlin, Germany. Patients were screened during January and August 2013 for inclusion criteria; these were defined as age of at least 18 years and current oral or transdermal opioid therapy for at least 4 weeks. Patients were excluded for analysis with evidence of comorbidity that significantly influenced stool frequency, eg patients with inflammatory bowel diseases.

Data were obtained from the electronic patient chart in the hospital mainframe computer and also based on the written documentation of clinical care in the study centers. Supervised chart review was performed by 2 investigators (FB, TB) using a consented electronic patient report form. Dosing of current opioid medication was transferred in oral morphine equivalents based on the German S3-guideline for Long-Term Opioid Use in Non-Cancer Pain 2014 (see also Table 1).^17,18^ During his first consultation, every patient provided information filling a standardized questionnaire adapted from the nationally recommended German pain questionnaire.^19^ This also addressed potential side effects of opioids. Constipation was rated from all patients in a self-assessment using a numeric rating Likert scale from 0 to 10 (Con-NRS, 0 “no problems” to 10 “worst possible symptom”). Additionally, symptoms of constipation were evaluated in a structured assessment based on ROME-III criteria serving as the gold standard to define constipation.^8^ This was performed by the attending pain specialists during the patient visit.

**TABLE 1:**
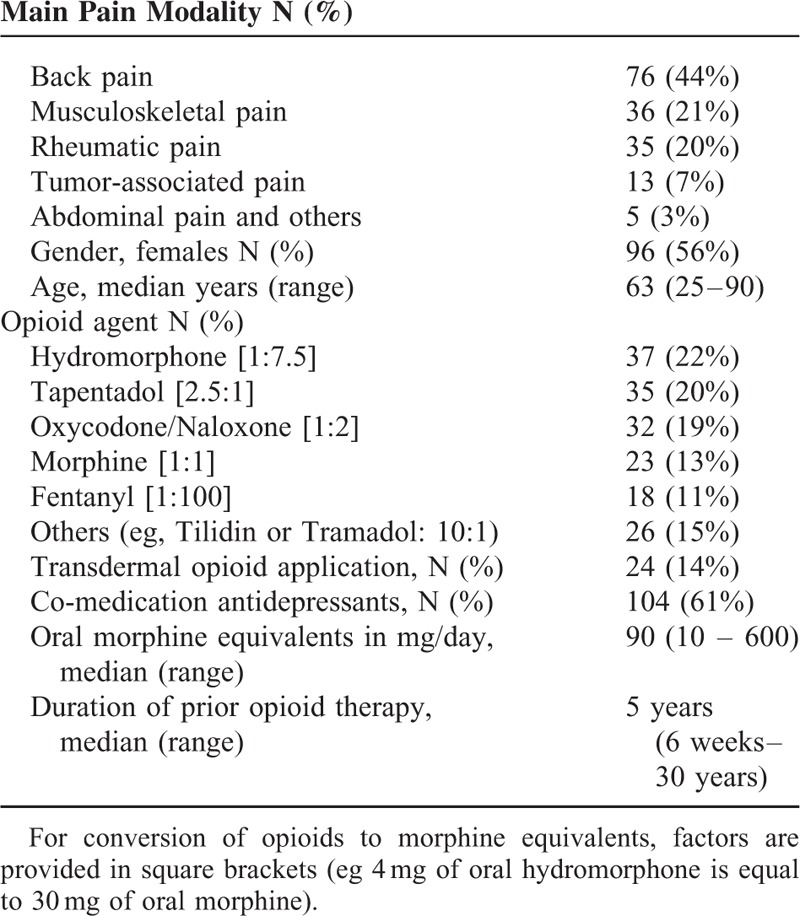
Basic Characteristics of Study Population (N = 171 Patients)

For statistical analyses SPSS version 22 was used. Descriptive data were summarized depending on the scale level and distribution using mean and standard deviation or median and range. For the evaluation of test performance, the area under the curve of the receiver-operating characteristic (AUC of ROC) was calculated. The rating scale was intended to be used as a screening instrument which requires maximized sensitivity. Consequently, based on results of the ROC analysis, the optimal cut-off with maximized sensitivity was searched. For this test value, sensitivity, specificity, positive predictive values, and negative predictive values were calculated. Con-NRS could also be of value to assess symptom severity from the subjective patient perspective. Therefore, a correlation between the summed number of symptoms of ROME-III items and the numeric rating scale was calculated and assessed by Kendall's tau. Additionally, logistic regression analysis was performed. For statistical significance analyses a 2-sided alpha level of <5% was defined.

This study was approved by the institutional review board of Charité university hospital and the local data safety board. Due to the observational nature of the trial the review board waived the need for informed consent.

## RESULTS

**FIGURE 1:**
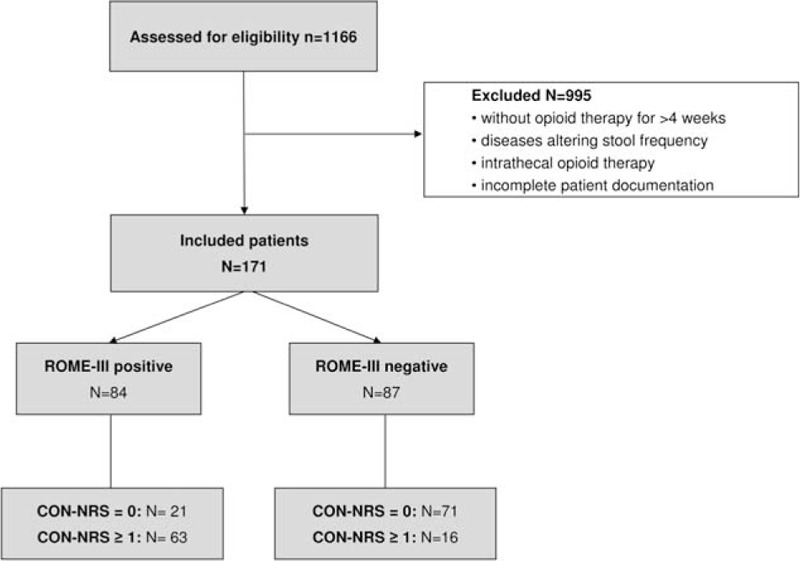
Flow chart for patient inclusion. Con-NRS – numeric rating scale from 0-10 for constipation; ROME-III – criteria for constipation: patients are diagnosed with constipation when fulfilling two or more of the criteria (frequency of defecations (<3 per week), straining during defecation, lumpy or hard stools, sensations of incomplete evacuation or of anorectal obstruction and, whether manual manoeuvres are required or laxative use for symptom control).^8.^

During the study period 1166 patients were screened with 171 fulfilling the inclusion criteria. Most patients were excluded as opioid therapy was not applied or prescribed for <4 weeks or history of diseases influenced stool frequency (Fig. 1).

Baseline characteristics of included patients are displayed in Table 1. Altogether 56% of the study population was women; median age was 63 years. Most common diagnoses for presentation were chronic back pain, musculoskeletal pain, or neuropathic pain.

Based on ROME-III criteria, 24 patients reported on 2 or more symptoms of constipation (14%), and 60 patients achieved sufficient symptom control using laxatives (35%). The remaining 87 patients (51%) did not fulfill the criteria of ROME-III for functional constipation. Interestingly, 39 of these 87 patients reported on episodes of constipation in their past medical history that was currently not present. Risk for constipation was significantly associated with increasing dose of morphine equivalents resulting in an OR of 1.065 (95% confidence interval 1.023–1.109 for increasing morphine equivalence dose by 10 mg; *P* = 0.002).

### Self-Reported Constipation (Con-NRS) and Test Characteristics

Altogether 92 patients reported a Con-NRS of 0 (53.8%), whereas 20 patients scaled their current Con-NRS with ≥8 points (11.7%). When dichotomizing the study population according to ROME-III criteria mean Con-NRS differed significantly between groups. Median Con-NRS in ROME-III negative patients was 0 (range 0–8) compared with 3.5 (range 0–10) in Rome-III positive patients (*P* < 0.001).

**FIGURE 2:**
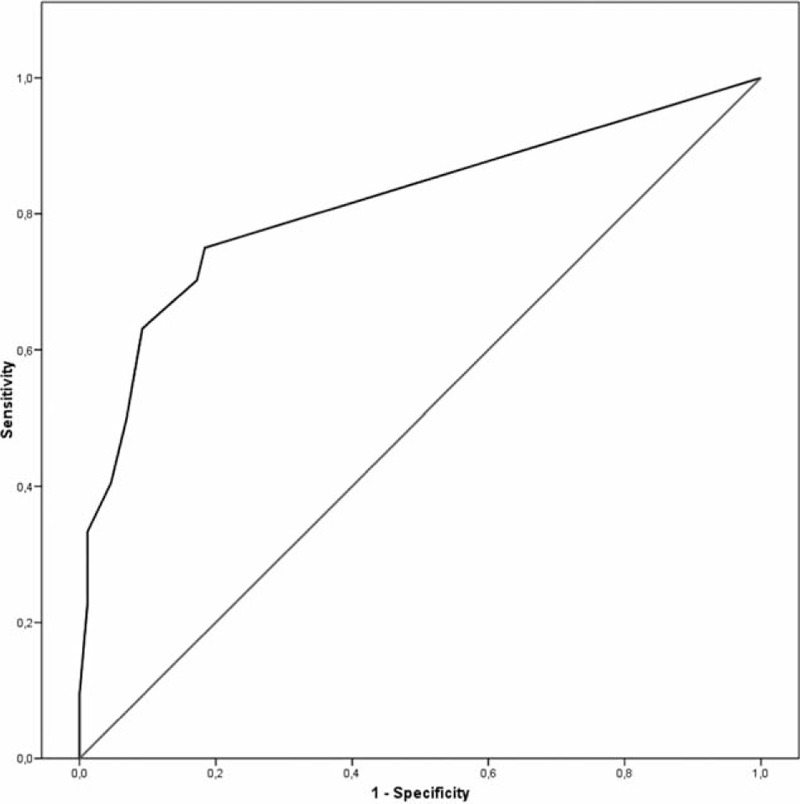
Evaluation of test performance of Con- NRS with Rome-III criteria as gold standard, receiver operating characteristic with area under the curve of 0.814 (95% confidence interval 0.748 – 0.880, p<0.001). Patients are diagnosed with constipation when fulfilling two or more of the ROME-III – criteria for constipation: patients are diagnosed with constipation when fulfilling two or more of the criteria (frequency of defecations (<3 per week), straining during defecation, lumpy or hard stools, sensations of incomplete evacuation or of anorectal obstruction and, whether manual manoeuvres are required or laxative use for symptom control).^8.^

To assess test characteristics for Con-NRS in the study population, patients were grouped according to ROME-III criteria as the gold standard for diagnosis of constipation. The corresponding receiver-operating characteristic of Con-NRS achieved an area under the curve of 0.814 (AUC 95% confidence interval 0.748 – 0.880, *P* < 0.001; Fig. 2).

With the intention to use Con-NRS as a screening instrument, the sensitivity of the test should be optimized to reduce false-negative results. With a threshold of Con-NRS ≥ 1, sensitivity and specificity achieved 79.7% and 77.2%, respectively. Here, the positive predictive value and negative predictive value achieved 70.3% and 81.6%, respectively. Notably, 21 patients (12.3%) in this study population did report a Con-NRS of 0 but fulfilled ROME-III criteria for constipation.

Additionally, we observed a positive significant correlation between number of ROME-III symptoms and Con-NRS with a correlation coefficient of 0.751 (*P* < 0.01).

## DISCUSSION

In this study including patients with long-term opioid medication a simple 11-item self-assessment scale for constipation achieved a sensitivity of ∼80% and moderate test performance. Consequently, the scale may be useful as an assessment tool to facilitate an individualized patient decision process in the management of potential medication-associated side effects. Notably, 21 patients were not identified with this instrument. The complex constellation of alternating and long-lasting symptoms of constipation might not be detected with this screening instrument entirely. Even though gastrointestinal symptoms are common side effects of opioids, it might be conflicting for patients to report such symptoms. Consequently, some patients may minimize these problems. As opioid-associated constipation occurs in a large number of patients once during treatment, patient education is highly important in every patient contact. The prevalence of constipation was 49% out of 171 patients: 14% of patients reported symptoms of constipation despite laxative use and altogether 35% of patients reported sufficient symptom control with laxatives. Constipation as a side effect of opioid therapy is well known. Most notably, patients on opioid therapy are typically not developing tolerance against this gastrointestinal side effect over time.^20^ It is necessary to address this issue in every patient with long-term opioids for continued patient education and participation.^21^ Finally, some authors concluded that constipation is inadequately assessed and often underdiagnosed.^16^

Against this background, a simple and feasible screening instrument could support patient management but should be able to sufficiently identify patients. Diagnostic screening instruments need to be tested in different settings to allow an estimate of diagnostic precision. For example, Rhondali et al evaluated self-reported constipation with a numeric 11-item scale in patients with advanced cancer and found a similar sensitivity of 84% for a cut-off ≥3 and a specificity of 62%.^16^ Notably, a false-negative rate of 16% was observed. These authors also reported that using a single binary question of constipation (yes/no) had a very high false-negative rate of one-third. Similarly, Noguera et al evaluated constipation with a numeric 11-item scale in palliative care patients and 66% of them treated with opioids. In this trial, the authors described a sensitivity of 83% and specificity of 63% for the cut-off ≥ 2.^15^ In line with our results, a significant number of patients were not detected with this numeric rating scale by Noguera et al. The authors had to conclude that this instrument is not sensitive enough to diagnose constipation. Unfortunately, validity of ROME-III criteria was not perfect in specific populations: in the study of Digesu et al 56 female patients reported on constipation but only 14% of them were identified by ROME-III criteria.^22^ Especially the quantification of symptoms in the Rome-III criteria is sometimes difficult for patients and may be 1 reason for diagnostic imprecision.

On the other hand, the use of a numeric rating scale may provide additional information regarding symptom severity or associated emotional strain. Exemplarily, Kindler et al reported that the visual analog scale for pain also correlated with anxiety or concern of patients.^23^ Repetitive measurements of a scale could inform more detailed about therapeutic success and allow evaluation of therapeutic measures. This aspect could be evaluated in further trials.

For clinical care in the context of long-term opioid therapy, medication-induced constipation is a complication with a variety of individual signs and symptoms and should be evaluated in the trustful consultation of patients with their physicians. Screening instruments may support patient management but cannot substitute an individualized risk assessment for constipation and other opioid-related side effects.

## LIMITATION

This observational trial evaluated a limited number of patients in the specific setting of specialized university hospital affiliated pain centers. Due to the retrospective nature of the study it is impossible to infer on causalities and data are limited to observed variables. Exemplarily, duration of constipation was not assessed. In contrast, this study evaluated the numeric constipation scale in a setting where it is intended to be used and not in an artificial setting of a prospective clinical study. The latter might increase external validity and larger data sets in similar setting should be studied to validate the findings.
